# Effect of Ce Content on Modification Behavior of Inclusions and Corrosion Resistance of 316L Stainless Steel

**DOI:** 10.3390/ma18010069

**Published:** 2024-12-27

**Authors:** Lei Zhao, Jichun Yang, Xiaoyang Fu

**Affiliations:** 1 School of Rare Earth Industry (School of Rare Earth Engineering and Technology), Inner Mongolia University of Science and Technology, Baotou 014010, China; leizhao1989@stu.imust.edu.cn (L.Z.); fxy9195@163.com (X.F.); 2 Key Laboratory of Green Extraction and Application of Light Rare Earth Resources of the Ministry of Education, Inner Mongolia University of Science and Technology, Baotou 014010, China

**Keywords:** rare earth Ce, 316L stainless steel, modification of MnS inclusions, decay resistance

## Abstract

The changes in the inclusions in 316L stainless steel before and after Ce addition were studied by adding different contents of Ce. The effects of rare earth Ce treatment on the modification of MnS inclusions in steel and the pitting corrosion resistance of 316L stainless steel are studied by field-emission scanning electron microscopy, laser confocal microscopy, the 6% FeCl_3_ corrosion weight loss test, and Tafel polarization curve test. The results show that the addition of Ce reduces the corrosion rate of stainless steel in 6% FeCl_3_ solution, and reduces the number and size of corrosion pits. The corrosion resistance is the best at a 0.0082% Ce content. In addition, the addition of Ce reduced the corrosion current density of stainless steel in 3.5% NaCl solution and increased the corrosion potential. The corrosion potential increased from −329 mV to −31.4 mV. Through Ce treatment, the grain is refined and the inclusions in the experimental steel are modified. With the increase in rare earth content, Mn S gradually transforms into Ce_2_O_2_ S inclusions. The morphology of the inclusions gradually change from the original long strips to a spherical shape, and the average size is significantly reduced, which improves the corrosion resistance of the stainless steel. The addition of rare earth Ce plays modifies the inclusions and purifies molten steel.

## 1. Introduction

With the deepening of global industrialization, there is an increasing demand for materials with certain properties, especially corrosion resistance [1,2]. 316L stainless steel is widely used in many fields such as offshore platforms [3,4] and in the petrochemical [5] and biomedical [6] industries because of its excellent corrosion resistance. However, in a corrosive environment, stainless steel is vulnerable to pitting corrosion. Once this phenomenon occurs, it seriously damages the overall performance of the material and eventually leads to material failure [7]. The corrosion resistance of steel is often affected by inclusions. Inclusions are unavoidable metallurgical defects in steel. Under the erosion of corrosive media, the initiation and development of pitting corrosion are often induced, which seriously affects the corrosion resistance of materials [8]. Inclusions are the main source of defects and corrosion. These substances are mainly sulfides, and there is a certain correlation of their size, shape, and composition with corrosion. Among them, MnS inclusions are often the sources of cracks in steel, thus affecting the corrosion resistance of steel [9]. Spherical inclusions help to reduce the formation of micro-cracks, improve the fusion performance with the steel matrix, and improve the corrosion resistance of steel [10]. Therefore, it is very important to study the regulation or modification technology of inclusions to inhibit the corrosion of stainless steel.

The addition of Cu to 316L stainless steel had no significant effect on the microstructure with complete austenite characteristics. At the same time, the pitting potential and protection potential of the solution-treated 316L stainless steel containing Cu in 0.9 wt % NaCl solution increased with the increase in Cu content but decreased gradually after aging, which was due to the high density of Cu-rich precipitation [11]. Studies have shown that the addition of rare earth elements can modify inclusions [12], purify molten steel [13], increase corrosion voltage, and reduce corrosion current density [14], thereby enhancing the corrosion resistance of steel. Liu et al. [15] pointed out that rare earth Ce can purify molten steel, reduce the corrosion current density, and improve the corrosion resistance of UNS S31803 stainless steel. Zhang et al. [16] reported that the addition of 0.0050~0.020% Ce to 2101 duplex stainless steel significantly improved the corrosion resistance and mechanical properties of the material. Specifically, as the corrosion potential increases, the inclusions change from Al_2_O_3_ and MnS to rare earth inclusions, and the tensile and impact properties are improved. Zhang et al. [14] pointed out that the addition of 0.0013~0.0019% Ce can refine the grain size and significantly improve the corrosion resistance of EH420 steel. Feng et al. [17] found that the addition of 0.0008~0.0042% Ce could transform MnS and other inclusions in steel into spherical rare earth inclusions, reduce the stress concentration of steel, improve the fusion with steel matrix, and thus improve the tensile and impact mechanical properties of U75V steel. According to these studies, a Ce content in the range of 0.0023% to 0.0082% wt. % can effectively refine the grains, improve the morphology of inclusions, improve the corrosion and mechanical properties of the material, maximize the material properties, and maintain cost-effectiveness in practical applications.

Although there have been relevant studies, few studies have been conducted on the corrosion rate, macroscopic morphology, microscopic morphology, and inclusions of 316L stainless steel with different rare earth Ce contents. At the same time, the effect of the rare earth element Ce on the modification of the inclusions in 316L stainless steel is not clear, and its mechanism still needs to be further studied. Therefore, in this study, 316L stainless steel was selected as the research object. The effects of the addition of rare earth Ce on the corrosion resistance of 316L stainless steel were studied by various means such as the 6 wt. % FeCl_3_ immersion test, laser confocal microscopy, electrochemical test, and SEM morphology and composition analyses. Through the characterization and analysis of inclusions in steel after adding rare earth Ce, the mechanism of metamorphic inclusions in 316L stainless steel is deeply explored, and then the influence of inclusions on its corrosion resistance is judged, which provides a theoretical reference for the subsequent research and development of high-performance stainless steel.

## 2. Experimental Materials and Methods

### 2.1. Preparation of Experimental Materials

In this study, the experimental steel was based on 316L stainless steel; Ce iron was added to it, then smelted in a medium-frequency vacuum induction furnace; and nitrogen was used as protective gas. The materials required for smelting included pure industrial iron, polycrystalline silicon, electrolytic manganese, ferromolybdenum, pure nickel, pure chromium, pure copper, and Ce ferroalloy (mass fraction, Ce content 30%), which were used as test steel raw materials. Cerium-iron alloy was added by secondary feeding, and the raw material composition is shown in Table 1. The ingot was heated to 1200 °C in a muffle furnace and then forged into 30 mm billets after holding for 2 h. The billets were treated with a solid solution at 1050 °C. The chemical composition of the experimental steel is shown in Table 2.

### 2.2. Experimental Methods

In order to observe the change in the inclusions in the steel with different cerium contents, the experimental steel was cut into 10 mm × 10 mm × 7 mm pieces, and the 10 mm × 10 mm plane was polished. GAIA 3 XMN (TESCAN, Brno, Czech Republic) field emission scanning electron microscope was used to observe and analyze the inclusions in steel by secondary electron (SE) and backscattered electron (BSE) modes under the conditions of accelerating voltage of 20 kV and working distance of 8.4~10.1 mm.

The initial mass of the steel sample was recorded with a 0.1 mg precision balance. Then, according to the ASTMG48 standard [18], 100 g FeCl_3_⸱6H_2_O was dissolved in 100 mL of deionized water to prepare a 6 wt. % solution. The samples were immersed in the 6 wt. % FeCl_3_ solution for 8 h, 16 h, 24 h, and 72 h, and the temperature of the solution was held constant at 22 °C. After removal of the sample, the corrosion morphology was observed with a laser confocal microscope (OLS 4000), and then the sample was de-rusted with de-rusting solution (500 mL HCl + 500 mL H_2_O + 3–10 g hexamethylenetetramine). After rinsing with anhydrous ethanol and drying, the remaining mass was weighed with a 0.1 mg balance. Finally, the corrosion morphology after de-rusting was observed with a field-emission scanning electron microscope (FESEM). According to Formula (1), the corrosion rate of each sample was calculated.

(1) $$v=\frac{m_{0}-m_{1}}{s\cdot t}$$

In this formula, v represents the corrosion rate in g/(m^2^·h); m_0_ is the initial mass of the sample before corrosion, g; m_1_ is the mass after removing corrosion products after corrosion, g; s is the surface area of corrosion sample, m^2^; t is the length of soaking time, h.

The 10 mm × 10 mm × 7 mm sample was cold-inlaid, and its 10 mm × 10 mm plane was exposed. After fixing the Cu wire on the back, it was packed with epoxy resin. The exposed 10 mm × 10 mm working face was ground to 2000 mesh with sandpaper. After polishing, it was washed and dried. The encapsulated sample was immersed in 3.5% NaCl solution, and the model of the electrochemical workstation was an SI1280B. The three-electrode system was used for the electrochemical test, in which the sample was used as the working electrode, the platinum electrode was used as the auxiliary electrode, and the saturated calomel electrode (SCE) was used as the reference electrode. The electrolyte was 3.5 wt. % NaCl solution, the potential scanning range was −0.25 to 0.25 V, and the scanning rate was set to 0.167 mV/s.

## 3. Results and Discussion

### 3.1. Effect of Ce on Microstructure of Experimental Steel

**Figure 1:**
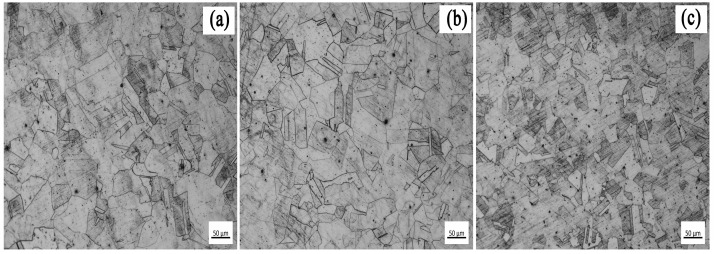
Microstructure of experimental steel with different Ce contents: (**a**) #1, (**b**) #2, (**c**) #3.

Figure 1 shows the microstructure of the experimental steel with different Ce contents. Under the same experimental conditions, the three kinds of test steel had a single-phase austenite structure. The austenite grains of steel #1 without rare earth Ce treatment were coarse and unevenly distributed. The austenite grains of the #2 experimental steel containing 0.0023% Ce were refined, their size was smaller, and their distribution was uniform. The grain size of the #3 experimental steel containing 0.0082% Ce was significantly smaller than that of #1 experimental steel, and the distribution is more uniform. It can be seen that with the increase in the rare earth Ce content, the austenite grain size obviously refined, and the distribution was more uniform; the experimental steel containing 0.0082% Ce performed best.

### 3.2. Effect of Ce on Inclusions in Experimental Steel

**Figure 2:**
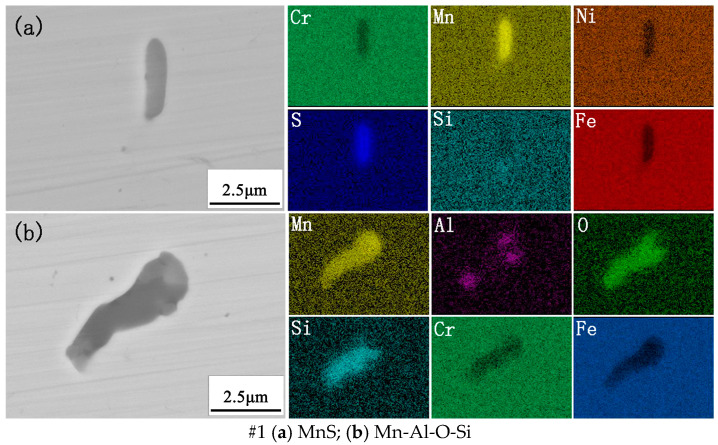

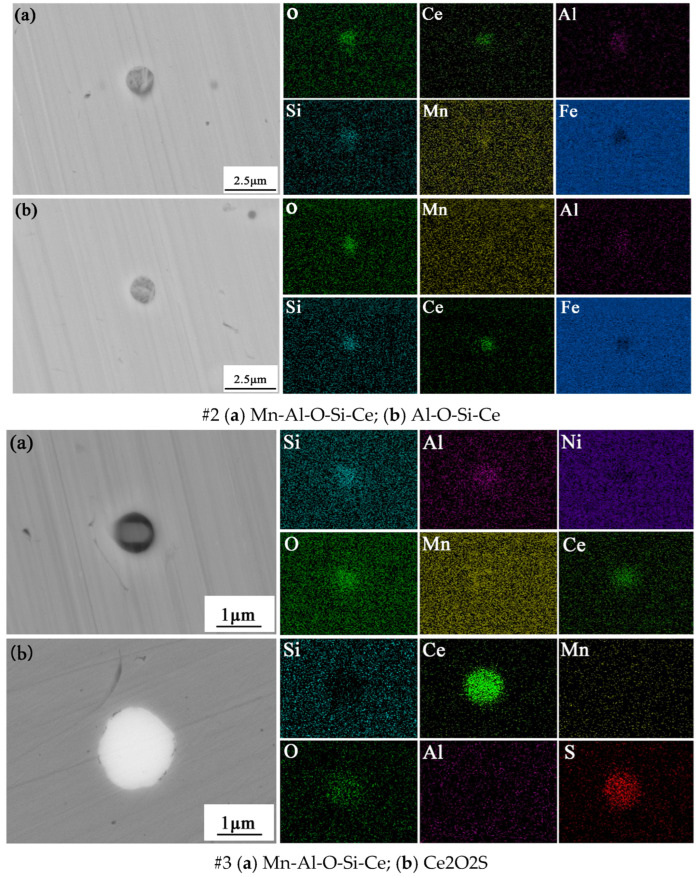
Morphology and composition of typical inclusions in experimental steel.

Figure 2 shows the morphology and composition of the typical inclusions in the experimental steel. There were two main types of inclusions in the #1 experimental steel. The morphology of the first type of inclusion was a long strip, which was mainly composed of Mn and S. Therefore, the first type of inclusion was MnS with a size of about 3.0 μm. The second type of inclusion was rod-shaped, which we found was Mn-Al-O-Si composite inclusions with a size of about 5.0–6.0 μm. After adding 0.0023% Ce, the inclusions in #2 steel were modified. The results showed that the first type of inclusion in #2 experimental steel was Mn-Al-O-Si-Ce composite inclusions modified by rare earth Ce, which were approximately circular in shape and about 1.5~2.0 μm in size. The second type of inclusion was Al-O-Si-Ce composite, which was spherical in shape and about 1.4 μm in size. After adding a 0.0082% mass fraction of Ce, the inclusions in #3 steel were fully modified. It can be seen from the figure that there were two main types of inclusions in the #3 experimental steel. The first type of inclusion was mainly composed of Mn-Al-O-Si-Ce composite inclusions, which were round in shape and about 1.0 μm in size. The second type of inclusion was spherical, the main component was Ce_2_O_2_S, and its size was significantly smaller than that of typical inclusions in #1 sample steel, at about 1.6 μm.

In summary, when the mass fraction of cerium in the experimental steel was 0.0023%, the inclusions in the steel were modified into Ce-containing composite inclusions, the morphology changed from the original long strips to the round, and the size reduced. When the mass fraction of cerium in the experimental steel was 0.0082%, the inclusions in the steel were modified to Mn-Al-O-Si-Ce and Ce_2_O_2_S inclusions. The size was obviously smaller than that of #1 steel without rare earth Ce, and the morphology changed from the original long strips to spherical in shape.

Studies have shown that rare earth Ce has the effect of modifying inclusions, which can transform MnS into rare earth inclusions or composite rare earth inclusions with a regular shape and smaller size, thereby enhancing the corrosion resistance of steel [10]. Lian et al. [19] reported that rare earth Ce and La can modify slender MnS into spherical rare earth inclusions, reduce the initiation and propagation of cracks, and improve the corrosion resistance of steel. This indicates that the formation of rare earth inclusions plays an active role in the corrosion resistance of steel.

### 3.3. The Effect of Ce on the Corrosion Rate of Experimental Steel

**Figure 3:**
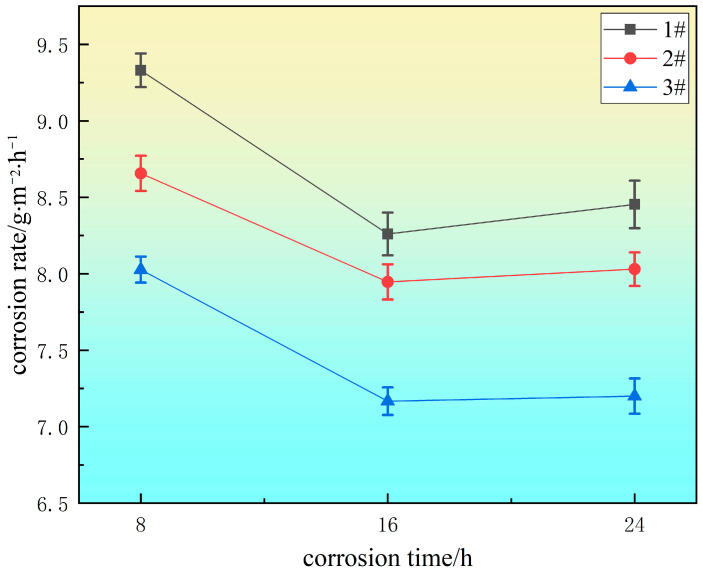
Effect of Ce on the corrosion rate of experimental steel.

Figure 3 is the corrosion rate diagram of the experimental steel after soaking for 8 h, 16 h, and 24 h. The corrosion rate of the experimental steel obviously changed with the amount of added Ce. In general, during the test period, the corrosion rate of the three groups of experimental steel decreased first and then increased with the increase in time. In the early stage of corrosion (within 16 h), the size of the corrosion pits on the surface of the sample was small. With the increase in corrosion time, the corrosion rate of the sample gradually decreased because the corrosion products covered the surface of the sample so that the corrosive ions could not pass through the corrosion rust layer and enter the matrix. Therefore, the trends in the changes in the corrosion rate of the three groups of experimental steels within 16 h were similar.

After more than 16 h, the pitting gradually expanded. With the increase in corrosion products, the cation concentration in the pits became larger. The H^+^ ions produced by cation hydrolysis reduced the pH in the pits, which led to the dissolution of the matrix around the inclusions and accelerated the corrosion rate. Among them, the corrosion rate of the #1 experimental steel without rare earth Ce increased rapidly, the corrosion rate of the #2 experimental steel increased gradually but less than that of the #1 experimental steel, and the corrosion rate of the #3 experimental steel increased slowly.

According to Section 3.4, due to the small pits at the initial stage of corrosion, the corrosion rates of the three groups of experimental steels had similar trends. After more than 16 h, with the increase in corrosion time, the MnS inclusions in #1 steel without rare earth Ce had poor bonding with the steel matrix, which led to large pitting holes. After the addition of rare earth Ce, the edge of the formed spherical rare earth inclusions was smooth, which improved the fusion with the steel matrix and effectively slowed the corrosion rate. Therefore, the effect of rare earth Ce in the corrosion enlargement stage was more significant.

### 3.4. The Effect of Ce on the Corrosion Morphology of the Experimental Steel

**Figure 4:**
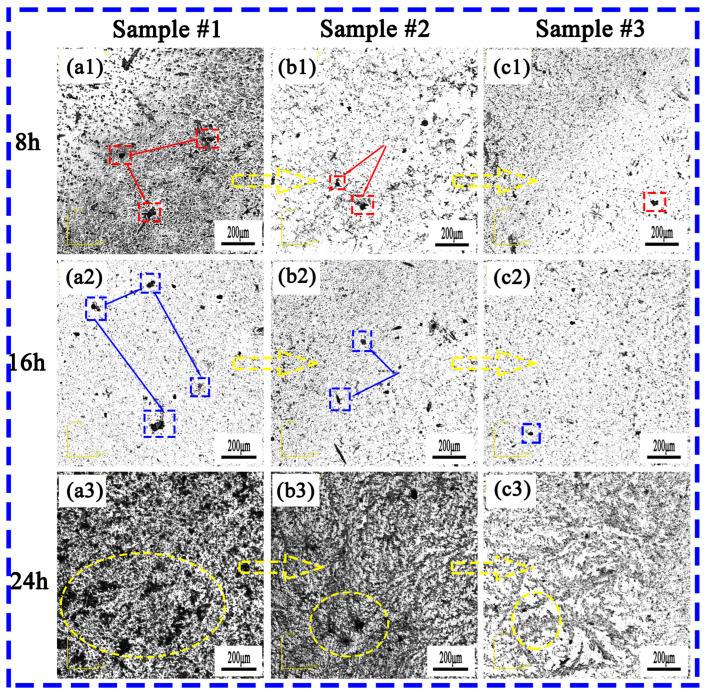
The surface morphology of the experimental steel after soaking in 6% FeCl_3_ solution for 8 h, 16 h, and 24 h: (**a1**–**a3**) #1; (**b1**–**b3**) #2; (**c1**–**c3**) #3. The red box represents the change of pitting corrosion on the surface of #1, #2 and #3 experimental steel after soaking in 6 % FeCl_3_ solution for 8 h. The blue box represents the change of pitting corrosion on the surface of #1, #2 and #3 experimental steel after soaking in 6 % FeCl_3_ solution for 16 h. The yellow circle represents the change of pitting corrosion on the surface of #1, #2 and #3 experimental steel after soaking in 6 % FeCl_3_ solution for 24 h.

Figure 4 shows the surface morphology of the three groups of experimental steel after soaking in 6% FeCl_3_ corrosion solution for 8 h, 16 h, and 24 h. In the early stage of corrosion, obvious pitting appeared on the surface of the #1 experimental steel. With the extension of the corrosion time, the surface of the steel substrate gradually became rough, loose, and porous, and the number and area of the pits gradually increased. This was mainly because the corrosion products became loose, resulting in the Cl^-^ in the corrosion solution easily passing through the corrosion products into the steel matrix, resulting in large corrosion pits in the matrix. The corrosion pits in the #2 experimental steel were improved compared with those of the #1 steel, and the diameter of the corrosion pits was smaller. The corrosion of the #3 experimental steel was not obvious in the initial stage. Compared with #2 steel, the diameter of the corrosion pits was smaller, the degree of corrosion was shallow, and most of the pits were small. In addition, under the same corrosion time, the corrosion pit diameters of the #2 and #3 experimental steel containing rare earth Ce were smaller than that of #1 steel without rare earth Ce, and the corrosion pit diameter and number of the #3 experimental steel was significantly smaller than those of the #1 and #2 experimental steel. Therefore, with the increase in rare earth Ce content, the corrosion resistance of the experimental steel also enhanced accordingly.

**Figure 5:**
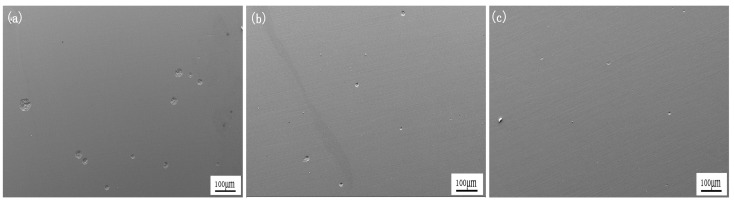
Surface morphology of the steel matrix of the experimental steel after removing rust by immersion in 6% FeCl_3_ solution for 24 h: (**a**) #1, (**b**) #2, (**c**) #3.

**Figure 6:**
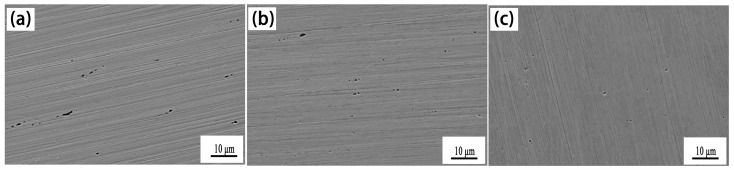
The micro corrosion morphology of the experimental steel immersed in 6% FeCl_3_ solution for 24 h after de-rusting: (**a**) #1, (**b**) #2, (**c**) #3.

Figure 5 and Figure 6 show the macro and micro morphologies of #1, #2, and #3 experimental steel immersed in 6% FeCl_3_ solution for 24 h after rust removal. It can be seen from Figure 5a that a large number of corrosion pits with a large size appeared on the surface of the #1 experimental steel. Figure 6a shows the micro corrosion morphology of the #1 experimental steel after rust removal, where long strips of corrosion pits are clearly visible. These corrosion pits were mainly due to the formation of pitting caused by MnS inclusions. The H^+^ generated by the hydrolysis of the cations in the pits reduced the pH, further causing the matrix to dissolve, and finally forming a long strip of pits. It can be seen from Figure 5b and Figure 6b that compared with the #1 experimental steel, the number of pits on the surface of #2 experimental steel was significantly lower, the number of dense corrosion holes was small, and the diameter of the corrosion holes was also significantly reduced. It can be seen from Figure 5c and Figure 6c that the number and diameter of the corrosion holes in the #3 experimental steel were significantly lower than those in the #1 experimental steel. Therefore, the addition of rare earth Ce improved the corrosion resistance of the experimental steel, and the corrosion resistance increased with the increase in rare earth Ce content.

Inclusions have a direct impact on the formation of corrosion pits. Because of the poor bonding between MnS inclusions and steel matrix, the corrosive medium easily gathers at the junction of inclusions and steel matrix, thus accelerating the generation of pitting corrosion [20]. The non-metallic inclusion MnS is the main source of pitting corrosion in steel, which has an important influence on the corrosion resistance of stainless steel. A large number of studies have shown that the rare earth inclusions generated after rare earth treatment can effectively improve the corrosion resistance of steel [21].

**Figure 7:**
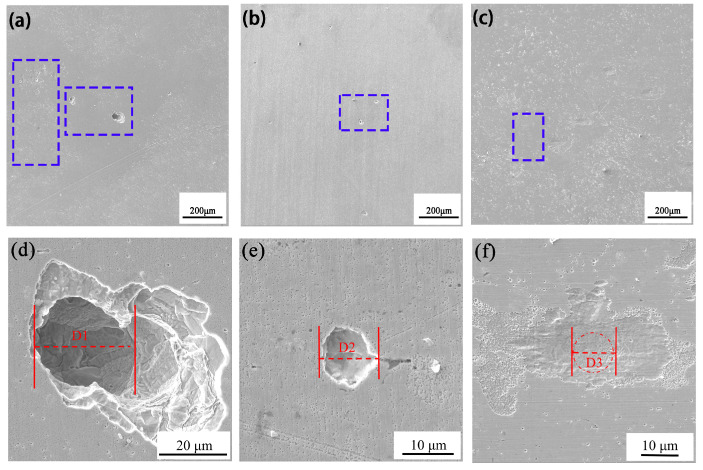
The surface morphology of the experimental steel after immersion in 6% FeCl_3_ solution for 72 h: (**a**,**d**) #1; (**b**,**e**) #2; (**c**,**f**) #3. The blue box represents the change of pitting corrosion on the surface of #1, #2 and #3 experimental steel after soaking in 6 % FeCl_3_ solution for 72 h.

**Figure 8:**
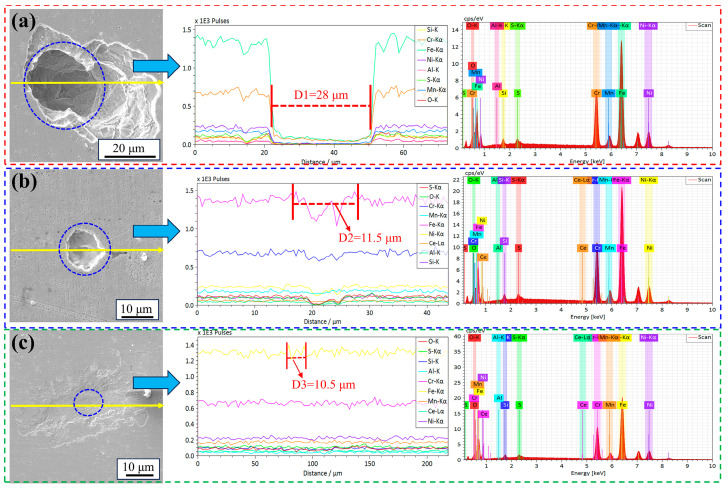
The pitting size and EDS spectrum of the experimental steel after soaking in 6% FeCl_3_ solution for 72 h: (**a**) #1; (**b**) #2; (**c**) #3.

Figure 7 and Figure 8 and Table 3 show the morphology and EDS of the pits of the #1, #2, and #3 experimental steel immersed in 6% FeCl_3_ solution for 72 h. From the diagrams, it can be seen that the number of corrosion pits on the surface of the #1 experimental steel was large, the shape of the corrosion pits was long strips, and the edge of the corrosion pits was sharp. The inside of the corrosion pit was in the form of a deep cavity. The diameter of the corrosion pit was large, about 28 μm.

For #2 steel with 0.0023% Ce, the number of corrosion pits on the surface of the experimental steel decreased, and the corrosion pits were circular and shallow, with a diameter of about 11.5 μm. For the #3 steel with 0.0082% Ce, the number of corrosion pits on the surface of the experimental steel was significantly reduced, and only slight corrosion marks appeared. The preliminary statistics showed that the diameter of the deepest corrosion pit was about 10.5 μm.

### 3.5. Polarization Curve Analysis

**Figure 9:**
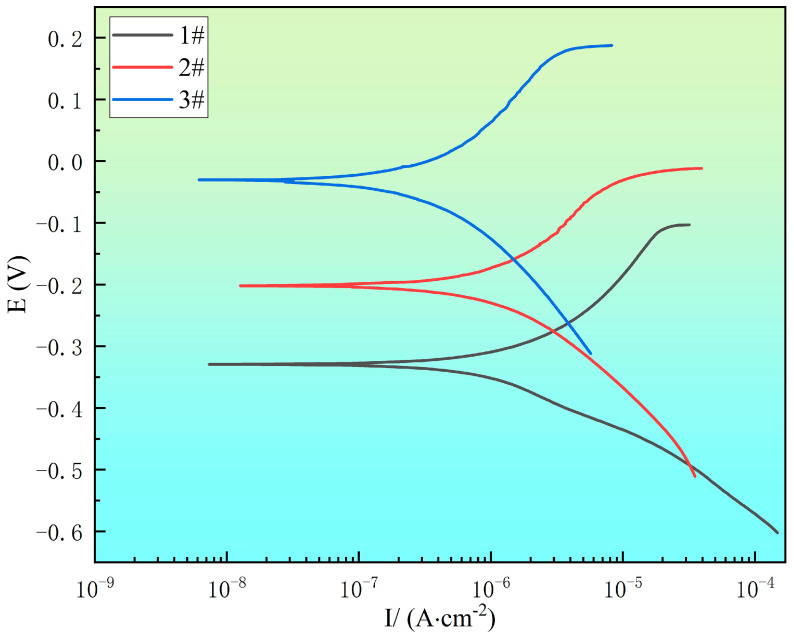
Polarization curve of experimental steel in 3.5% NaCl solution.

Figure 9 shows the polarization curves of the experimental steel with different rare earth Ce contents in 3.5% NaCl solution. At the same time, after fitting by the Tafel extrapolation method, the corresponding corrosion potential, pitting potential, and corrosion current density were as shown in Table 4. The current density of the #1 experimental steel was 3.07 × 10^−6^ A·cm^−2^, the current density of the #2 experimental steel was 2.13 × 10^−6^ A·cm^−2^, and the corrosion current density of the #3 experimental steel was 1.71 × 10^−6^ A·cm^−2^. The corrosion current density of the #3 experimental steel was the lowest; the pitting potentials of #1~#3 were −104.9, −17.5, and 184.3 m V, respectively. The pitting potential of the #3 experimental steel was the highest. The corrosion potentials of #1–#3 were −329, −202, and −31.4 m V, respectively. The corrosion potential of the #2 and #3 experimental steel was significantly higher than that of the #1 experimental steel.

The above experimental data show that the corrosion resistance of stainless steel without rare earth is poor. The main reason is that MnS inclusions and composite oxide inclusions have angular tips, which easily concentrate stress concentration, resulting in poor bonding with the steel matrix and the induction of matrix corrosion. At the same time, corrosion products are strongly corrosive, and the hydrolysis reaction of the cations produced by dissolution generates a large amount of H^+^, resulting in local acidification and aggravation of corrosion. The addition of rare earth Ce modifies the MnS inclusions into spherical rare earth inclusions, improves the fusion with the matrix, inhibits the effect of corrosion, improves the self-corrosion potential of stainless steel, and reduces its electrochemical corrosion rate. The comprehensive analysis of corrosion current density and corrosion voltage data showed that the addition of rare earth Ce significantly enhanced the corrosion resistance of the experimental steel, and with the increase in the Ce content, the improvement effect of corrosion resistance was more obvious. In this study, the #3 experimental steel with a rare earth Ce mass fraction of 0.0082% showed the best corrosion resistance.

### 3.6. Mechanism Analysis of Rare Earth Ce Improving the Corrosion Resistance of Stainless Steel

Non-metallic inclusions in steel, especially MnS, are the main source of pitting corrosion. Pitting corrosion is a type of localized corrosion in steel, which is highly destructive and concealed. The morphology and size of MnS inclusions have a direct effect on the properties of stainless steel.

During the corrosion process of stainless steel, MnS inclusions lead to galvanic corrosion due to their high activity and low potential, becoming the anode, while the steel matrix is the cathode. Pitting corrosion easily occurs with MnS inclusions. The S-containing ions produced by MnS dissolution interact with the Cl^−^ in the solution, resulting in pits around the inclusions. The concentration of cations in the pits around the inclusions increases, and the local acidification intensifies, which accelerate pitting corrosion. At the same time, MnS inclusions and composite oxide inclusions are prone to stress concentration at their corners, which further accelerates corrosion [22]. The addition of rare earth Ce can modify the inclusions, make the MnS inclusions spherical, improve the fusion with the matrix, and inhibit corrosion [23]. Spherical inclusions have a smaller surface area and more uniform distribution than irregular inclusions, which can effectively reduce strain concentration. This helps to improve the plasticity and toughness of the material, reduce the initiation and propagation of cracks, and thus improve the overall mechanical properties of the material. At the same time, as the cathode in the process of electrochemical corrosion, the rare earth inclusions have poor conductivity, which further inhibit corrosion.

Regarding the morphology of sulfides, long-strip-shaped MnS inclusions easily cause the pitting corrosion of stainless steel because such inclusions are prone to stress concentration at the tip and are more susceptible to stress, which promote pitting corrosion and accelerate the corrosion process. Shi et al. [24] pointed out that the thermal expansion coefficient of long-strip MnS inclusions is quite different from that of the steel matrix, leading to the formation micro-cracks and thus accelerating the corrosion process. The spherical rare earth inclusions have smooth edges, and corrosion pits do not easily form around them, thus improving the corrosion resistance of steel. Regarding the size of sulfide, generally speaking, the corrosion effect of small inclusions on steel is less than that of large inclusions [25]. In summary, the shape and size of MnS inclusions affect the pitting corrosion behavior of stainless steel. Spheroidization and small rare earth inclusions help improve corrosion resistance.

## 4. Conclusions

(1) The grain size was refined, and the inclusions in the experimental steel were improved with rare earth Ce treatment. With the increase in the rare earth content, MnS gradually transformed into Ce_2_O_2_ S inclusions. The morphology of the inclusions gradually changed from the original long strips to spherical in shape, and the average size was significantly reduced, which improved the corrosion resistance of the stainless steel. The rare earth element Ce modifies the inclusions of and purifies molten steel.

(2) The addition of Ce to 316L stainless steel reduced its corrosion rate in 6% FeCl_3_ solution and improved its corrosion resistance. With the increase in rare earth Ce content, the number and size of the corrosion pits significantly reduced, and the corrosion resistance of the experimental steel was enhanced. The experimental steel with a 0.0082% Ce content had the best performance.

(3) Stress easily concentrated in the stainless steel without rare earth due to the angular tip of MnS and the composite oxide inclusions, resulting in poor bonding with the steel matrix, leading to corrosion. After the addition of rare earth element Ce, the inclusions were spheroidized, the bonding with the steel matrix was improved, the corrosion was inhibited, and the electrochemical activity of the stainless steel in 3.5% NaCl solution was reduced, which was manifested through the positive movement of corrosion potential and the decrease in current density.

## Figures and Tables

**Table 1:** Raw materials used in experimental steel and their composition (wt. %).

Alloy Type	C	Si	Mn	S	P	Cu	Mo	Ni	Cr	Ce	Fe
Electrolytic manganese	0.0010	0.0014	99.933	0.032	0.0007						Bal.
Polysilicon		99.99									0.0003
Industrial pure iron	0.0031	0.0057	0.0052	0.0025	0.0037						Bal.
Ferromolybdenum	0.08	0.42		0.0860	0.0270		55.01				
Pure nickel								99.99			
Pure chromium									99.99		
Pure copper						99.99					
Cerium iron										30	Bal.

**Table 2:** Chemical composition of experimental steel (wt. %).

Steel Code	C	S	P	Si	Mn	Ni	Cr	Mo	Cu	Ce
#1	0.015	0.005	0.006	0.965	1.92	13.55	18.62	2.00	0.352	0
#2	0.013	0.004	0.005	0.962	1.92	13.57	18.71	2.05	0.351	0.0023
#3	0.016	0.005	0.006	0.961	1.89	13.50	18.69	2.06	0.354	0.0082

**Table 3:** Mass percentage of each element (wt. %).

Steel Code	Fe	Cr	Si	Al	S	Mn	Ni	O	Ce
#1	36.11	25.99	17.37	9.08	4.52	3.89	2.22	0.82	0
#2	35.62	24.96	17.71	8.58	4.44	3.50	1.53	0.51	3.16
#3	38.36	26.77	14.75	6.94	4.37	3.54	1.69	0.44	3.14

**Table 4:** Polarization curve fitting parameters of experimental steel in 3.5% NaCl solution.

Steel Code	E_corr_/mV	E_pit_/mV	J_corr_/(A·cm^−2^)
#1	−329	−104.9	3.07 × 10^−6^
#2	−202	−17.5	2.13 × 10^−6^
#3	−31.4	184.3	1.71 × 10^−6^

## Data Availability

The original contributions presented in this study are included in the article. Further inquiries can be directed to the corresponding author.
